# High-Fidelity Reprogrammed Human IPSCs Have a High Efficacy of DNA Repair and Resemble hESCs in Their MYC Transcriptional Signature

**DOI:** 10.1155/2016/3826249

**Published:** 2016-09-01

**Authors:** Pratik K. Nagaria, Carine Robert, Tea Soon Park, Jeffrey S. Huo, Elias T. Zambidis, Feyruz V. Rassool

**Affiliations:** ^1^Department of Radiation Oncology, University of Maryland School of Medicine, Baltimore, MD 21201, USA; ^2^Institute for Cell Engineering and Sidney Kimmel Comprehensive Cancer Center, The Johns Hopkins University School of Medicine, Baltimore, MD 21205, USA

## Abstract

Human induced pluripotent stem cells (hiPSCs) are reprogrammed from adult or progenitor somatic cells and must make substantial adaptations to ensure genomic stability in order to become “embryonic stem cell- (ESC-) like.” The DNA damage response (DDR) is critical for maintenance of such genomic integrity. Herein, we determined whether cell of origin and reprogramming method influence the DDR of hiPSCs. We demonstrate that hiPSCs derived from cord blood (CB) myeloid progenitors (i.e., CB-iPSC) via an efficient high-fidelity stromal-activated (sa) method closely resembled hESCs in DNA repair gene expression signature and irradiation-induced DDR, relative to hiPSCs generated from CB or fibroblasts via standard methods. Furthermore, sa-CB-iPSCs also more closely resembled hESCs in accuracy of nonhomologous end joining (NHEJ), DNA double-strand break (DSB) repair, and C-MYC transcriptional signatures, relative to standard hiPSCs. Our data suggests that hiPSCs derived via more efficient reprogramming methods possess more hESC-like activated MYC signatures and DDR signaling. Thus, an authentic MYC molecular signature may serve as an important biomarker in characterizing the genomic integrity in hiPSCs.

## 1. Introduction

Although human induced pluripotent stem cells (hiPSCs) resemble hESCs in many respects [1, 2], the therapeutic utility of hiPSCs is limited by low reprogramming efficiency [3–6] and poor genomic integrity [7–10]. A deeper understanding of the mechanisms that control these roadblocks will be vital for the use of hiPSCs in regenerative medicine. Reprogramming efficiency is controlled by intrinsic and extrinsic microenvironmental factors that are determined by the method employed [5]. Standard protocols often utilize inefficient and potentially mutagenic retroviral mediated transgene factor expression (e.g., OSKM:* OCT4*,* SOX2, KLF4,* and* C-MYC*, or OSNL, i.e.,* OCT4, SOX2, NANOG,* and* LIN28*) [11]. More clinically useful nonviral, nonintegrating methods have also been widely employed (e.g., plasmids, microRNA), albeit with a significantly reduced reprogramming efficiency [11]. The choice of somatic donor and* in vitro* microenvironmental conditions also significantly influences reprogramming efficiency. For example, we previously demonstrated that bone marrow stromal cell (MSC) activation robustly activated MYC complex-regulated genes of pluripotency that subsequently facilitated high-quality reprogramming of human myeloid progenitors (MP) differentiated from CD34^+^ hematopoietic stem-progenitor cells [12]. Activation of MYC-regulated factors potentially enhanced the rate and efficiency of reprogramming [13]. MYC may also play a key role in regulating promoters and microRNAs associated with core pluripotency-associated genes [14, 15]. These findings implicate targets of the MYC network not only in playing a key role in controlling the efficiency of reprogramming, but also in maintaining stem cell pluripotency.

Efficacious DNA double-strand break (DSB) repair is a key element in maintenance of high genomic integrity [16, 17]. In mammalian cells, homologous recombination repair (HR) provides precise, error-free DSB repair by using a homologous sister chromatid as a template for repair [18]. In contrast, repair by nonhomologous end joining (NHEJ) joins DNA ends directly and is thus prone to errors [19]. In hESCs, repair of DSBs occurs mainly by HR [17]. We and others have reported a form of DSB end-joining repair in hESCs that is relatively error-free [17, 20, 21]. However, overall DNA repair properties in reprogrammed cells are more heterogeneous than hESCs [22, 23]. For example, we previously demonstrated that hiPSCs derived from mesenchymal stem cells (MSCs) or fibroblasts were more deficient than hESCs in DSB end-joining capacity despite similarities in the precision of repair between them [20]. These studies suggest that efficient DSB repair properties confer an advantage in achieving completion of faithful reprogramming to an authentic hESC-like state [24]. However, the mechanisms that control efficient DSB repair during reprogramming are unclear.

MYC, which can associate with the E-box elements in the promoters of several DSB repair genes and can amplify the cell's transcriptional program by binding to promoter and enhancer elements, represents a strong candidate for regulation of DSB repair in pluripotent cells [25, 26]. Determining these mechanisms not only is critical in finding the most efficient way to derive iPSCs, but also can be applied to measures ensuring the safe clinical use of iPSCs with high genomic integrity. To address these questions, we evaluated previously reported human CB-derived sa-CB-iPSCs generated with high efficiencies (1–4% input cells) and compared them to CB- and fibroblast-derived hiPSCs derived via standard methods (<0.001–0.5% input cells) [27]. Our data reveal that in response to radiation-induced DNA damage, sa-CB-iPSCs possessed a DDR signature that more closely resembles that of hESCs. These sa-CB-iPSCs also possess lower baseline levels of endogenous DNA DSBs and a greater accuracy of DSB end-joining, compared to standard CB-iPSCs and fibroblast-iPSCs. Moreover, we show that C-MYC may play an important role in facilitating a stringent and high-fidelity DSB response in hESCs and hiPSCs. Collectively, our data suggest that more efficient activation of MYC-associated DDR signaling during reprogramming or DSB damage may enhance the genomic integrity of hiPSCs and increase their ultimate clinical utility.

## 2. Materials and Methods

*Ethics Statement (Human Embryonic Stem Cell Lines)*. All hESC lines used in this study were obtained commercially from the WiCell Research Institute (Wisconsin International Stem Cell Bank, WISCB). The use of all WISCB-donated hESC lines in these studies was approved by the Johns Hopkins Embryonic Stem Cell Research Oversight (JHU-ESCRO) Committee and the University of Maryland School of Medicine Embryonic Stem Cell Research Oversight Committee (UMSOM-ESCRO) and conforms strictly to standards of both institutions, including written informed consent. All experiments conducted in these studies also conformed to guidelines outlined for hESC and pluripotent stem cell research by the National Academy of Sciences and the National Institutes of Health (NIH).

### 2.1. Cell Culture

Pluripotent stem cells were routinely cultured on irradiated primary murine embryonic fibroblasts (MEF), derived from embryos of CF1 and DR4 F1 mice at embryonic days of 12.5 or 13.5 (P2/P3), or purchased from GlobalStem (Rockville, MD). Human pluripotent stem cell cultures were maintained in DMEM/F12 (Invitrogen) medium supplemented with 20% Knockout Serum Replacement (KOSR; Gibco), 0.1 mM MEM nonessential amino acids (Gibco), 1 mM L-glutamine (Gibco), 0.1 mM *β*-mercaptoethanol (Sigma-Aldrich, St. Louis, MO), and 4 ng/mL FGF2 (R&D Systems, Minneapolis, MN) at 37°C, 5% CO_2_, and 85% relative humidity. The medium was changed daily on hESCs and hiPSC cultures. For experiments, human pluripotent stem cells were first transitioned from MEF feeder layers onto a BD-Matrigel*™* (BD Biosciences) matrix precoated plate and cultured in mTESR1*™* medium (Stem Cell Technologies, Vancouver, Canada). The mTESR1 growth media were replenished daily. Purified (>95%) human CD34^+^ CB progenitors (also referred to as “starting CB progenitors”) from pooled donors were purchased from AllCells (Emeryville, CA) and cultured in the hematopoietic growth medium (HPGM).

### 2.2. Generation of Episomal hiPSCs

Detailed methods for generation and characterization of hiPSC lines were previously described [12, 28]. Details of hiPSC lines are provided in Table S1 in Supplementary Material available online at http://dx.doi.org/10.1155/2016/3826249. In brief, sa-CB-iPSC lines (CB6.2, 6.13, 19.11, and E12C1) were derived via nucleofection of stromal-activated CD34^+^ MP with 7 or 4 episomal factors (7F, SOKMNLT; SOX2, OCT4 (POU5F1), KLF4, c-MYC, NANOG, LIN28, and SV40L T antigen; 4F, SOKM) using the AMAXA II Nucleofector device (Lonza). Standard episomal CB-iPSC lines were derived without stromal activation with either four (4F; SOKM) or seven episomal factors (7F) from either CB-derived CD34^+^ MP (4F: E17C1, E20C2, and E24C1) or CB-derived unsorted mononuclear cells (7F: iCB9, iCB8, and iCB2.5) [29], kindly provided by Dr. Igor Slukvin (University of Wisconsin-Madison). Skin fibroblast-derived hiPSC line iHUF3, derived with four retroviral factors (SOKM), was previously described (Byrne et al.) and kindly provided by Dr. Renee Reijo-Pera (Stanford University) [27]. Requests for hiPSC lines should be addressed to Elias T. Zambidis (ezambid1@jhmi.edu).

### 2.3. Gene Expression Microarrays

**Figure 1:**
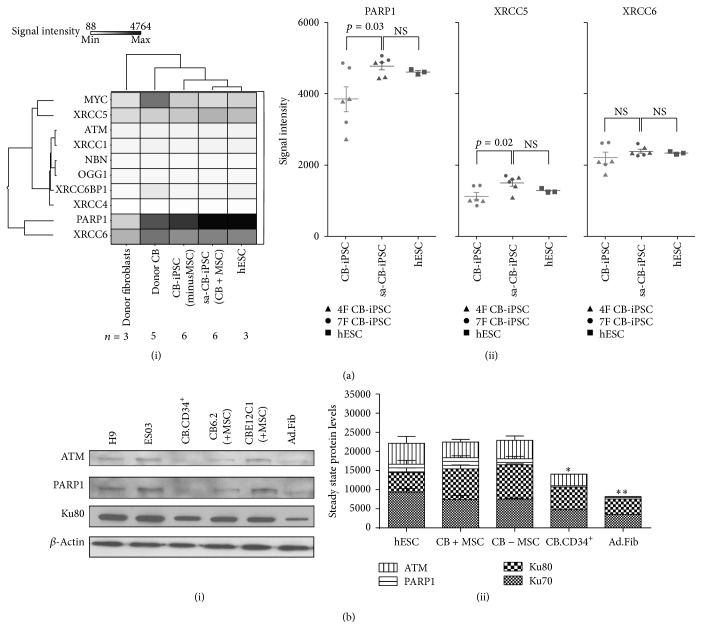
CB progenitors and CB-derived iPSCs closely resemble hESCs DNA repair gene expression signature. Microarray gene expression of selected DNA repair genes. (a)(i) Shown are hierarchical clustering heatmaps of mRNA from donor fibroblasts, donor CD34^+^ CB, and CB.iPSC derived with (+MSC) and without (−MSC) bone marrow stromal cell activation. hiPSC lines included sa-CBiPSC derived from stromal-activated CD34^+^ MP (*n* = 6; E5C3, E12C5, and E17C6: 6.2, 6.13, and 19.11), standard CB-iPSC, lines derived from CD34^+^ MP without stromal activation (*n* = 3, E17C1, E20C2, and E24C1), and standard CB-iPSC lines derived from CB unsorted mononuclear cells (*n* = 3, iCB9, iCB8, and iCB2.5). hESC lines included (*n* = 3) H9, H7, and ES03. Signal intensities are from averaged independent biological replicate microarray samples (*n* as indicated). Expression array data depicts normalized values of the mean transcript levels for a subset of DDR genes in each group of the indicated cell lines. (a)(ii) Dot plots represent the normalized values of the signal intensities for PARP1/XRCC5/XRCC6 with corresponding *p* values between categories indicated in the array data in (a)(i) (*n* as indicated in (a)(i)). Paired tests with significance *p* < 0.05 (*∗*) or without significance (NS; *p* > 0.05) with values of control hESC are indicated (*▲* = 4F CB.iPSC;* ●* = 7F CB.iPSC). (b)(i) Representative Western blot from the whole cell lysates of hESCs (H9 and ES03), CB (CD34^+^), two independent sa-CB-iPSCs (6.2 and E12C1), and adult fibroblasts (Ad.Fib) showing the steady state levels of PARP1 and Ku80 and ATM. *β*-Actin was used as the loading control. (b)(ii) Graphical representation of Western blots by ImageJ quantified-densitometry analysis normalized to *β*-actin (*n* = 3) in hESC (H9, ES03, and H7), sa-CB-iPSC (CB6.2, CB6.13, and CB19.11), standard CB mononuclear CB-iPSC (iCB9, iCB8, and iCB2.5), CB (CD34^+^), and adult fibroblasts (Ad.Fib). Results are representative of the mean of two independent experiments of each set ± SEM, ^*∗*^ *p* < 0.05 and ^*∗∗*^ *p* < 0.01, based on 2-way ANOVA (multiple comparisons test) on combined expression of genes.

Details of the microarray analysis were described before [12]. Human HT-12 Expression BeadChip arrays (Illumina, San Diego, CA) were used for microarray hybridization to examine the global gene expression of hESC, hiPSC, and starting populations (CD34^+^ progenitors and fibroblasts). The NIH Gene Expression Omnibus has issued the accession numbers GSE44425 (Figure 1, Figure 5(c)) for the deposited microarray data related to the above manuscript.

### 2.4. DNA Damage, Apoptosis, and MYC Inhibition Studies

For irradiation (IR) studies, pluripotent stem cells were exposed to X-ray radiation using a Pantak HF320 X-Ray machine (250 kV peak, 13 mA; half-value layer, 1.65 mm copper) at a dose rate of 2.4 Gy/min. For experiments involving MYC inhibitor (10058-F4, Sigma-Aldrich, St. Louis, MO), the cells were treated with either the control solvent (DMSO) or the drug at dose of 50 *μ*M for 24 h before X-ray IR. Following 24 h treatment, the medium was replaced before exposure to IR. For knockdown studies, siMYC (ON-TARGETplus*™*, Dharmacon, Thermo Fisher Scientific) was utilized. The cells were transfected with siMYC (2 *μ*g) using Lipofectamine*™* 2000 (Life Technologies), 48 h before exposure to IR.

### 2.5. Whole Cell Extracts and Nuclear Extracts

Whole cell extracts were prepared with lysis buffer (25 mM Tris-HCl (pH 7.5), 333 mM KCl, 1.3 mM EDTA, and 4 mM DTT) with protease inhibitor cocktail (Roche, Branchburg, NJ) and phosphatase inhibitors cocktail (Sigma-Aldrich). Nuclear extracts were prepared using the CelLytic Nuclear Extraction Kit (NEXTRACT*™*, Sigma-Aldrich) without the use of any detergents. The nuclear extracts used for the DNA repair assay were dialyzed against the E-buffer (20 mM Tris-HCl (pH 8.0), 20% glycerol, 0.1 M K(OAc), 0.5 mM EDTA, and 1 mM DTT).

### 2.6. Immunoblotting Analysis

20 *μ*g of proteins was separated by electrophoresis through either 4–10% or 4–15% polyacrylamide gradient gels (Mini-PROTEAN TGX) (Bio-Rad Laboratories, Hercules, CA) and then transferred to PVDF membranes (Thermo Fisher Scientific, Waltham, MA). After blocking, membranes were probed with primary antibodies mouse Ku70 (1 : 500, E-5, SC17789, Santa Cruz Biotech (SCB), Dallas, TX), Ku80 (Calbiochem, EMD Millipore NA54), PARP1 (1 : 2000, CS # 9532, Cell Signaling, Beverly, MA), p53 (1 : 1000, CS # 9282), pATM (1 : 1000, Millipore, Billerica, MA), *γ*H2AX (Millipore, Clone JBW301, 05-636), *β*-actin (1 : 5000, Sigma-Aldrich), and *β*-tubulin (CS # 2128) as loading controls. After probing with adequate secondary antibodies (anti-mouse IgG-CS and anti-rabbit, BioLegend, San Diego, CA), proteins expression was detected using enhanced chemiluminescence (ECL; 100 mM Tris-HCl (pH 8.5), luminol, coumaric acid, and hydrogen peroxide).

### 2.7. *In Vitro* NHEJ Assays (Plasmid Reactivation: PUC18 and EJ5-ISce1)

We used the DNA repair fidelity assay (PUC18-based) as described before [30]. For the assay, 2 *μ*g of EcoRI linearized PUC18 was incubated with 4 *μ*g of nuclear extract. Reactions (in 20 *μ*L volume) were carried out in ligation buffer (50 mM triethanolamine-HCl (pH 7.5), 60 mM KOAc, 50 *μ*M deoxynucleotide triphosphates, 2 mM ATP, 1 mM DTT, and 100 *μ*g/mL BSA). The mixture was incubated for 16 h at 18°C. Following the incubation, 10 ng of purified plasmid DNA was used to transfect* Escherichia coli* strain DH5*α*. Transformed cells were plated on LB agar plates, including 100 *μ*g/mL carbenicillin, 20 mg/mL X-gal, and 200 mg/mL isopropyl-1-thio-*β*-d-galactopyranoside. To allow for spontaneous rejoining/incomplete EcoRI cutting, assay controls were conducted without nuclear extract. In addition, to correct for bacterial plating numbers and determine whether nuclease activity was affecting plasmid efficacy, cells were plated on Luria-Bertani agar without carbenicillin.

For the EJ5-*Isce1 *assay, we used a protocol adapted from the one designed by Bennardo and colleagues but modified for* in vitro* plasmid reactivation analysis [31]. Briefly, the pimEJ5GFP reporter plasmid (Addgene Plasmid 44026) [31] was enzymatically linearized with* I-Sce1* (New England Biolabs (NEB), Ipswich, MA) at 37°C overnight. Linearized plasmid was dephosphorylated using Shrimp Alkaline phosphatase (SAP) (NEB), and column 500 ng DNA was incubated with dialyzed nuclear extracts (2 *μ*g) of respective cell lines, and ligation reactions were performed in ligation buffer (10x T4 ligase buffer, 2 mM ATP, and 50 *μ*M deoxynucleotide triphosphates). Following* in vitro *ligation, the plasmid DNA was column-purified and GFP genes were PCR-amplified using the primers p1 (Fwd) 5′-CTGCTAACCATGTTCATGCC-3′ and p2 (Rev) 5′-AAGTCGTGCTGCTTCATGTG-3′, as described by Bennardo et al. [31]. Following the PCR, we redigested plasmid with* I-Sce1* to differentiate between NHEJ repair that was completed with* I-Sce1* restoration (S+) and repair completed with loss of* I-Sce1* site (i.e., “S−” with deletions). Undigested and digested PCR products were fractionated on 2% agarose and visualized with the GelStar*™* Nucleic Acid Stain (Lonza). S-fragment was excised from the gel and cloned into PCR2.1® TOPO (Life Technologies). Cloned products were transformed into OneShot® TOP10 chemically competent cells (Life Technologies) and plated on LB plates with kanamycin resistance. DNA from 5 colonies from each experiment was sequenced using the M13 primers. A total of 15 colonies were analyzed from three independent experiments, and TOPO plasmids were sequenced at the UMB TGL/Biopolymer core facility.

## 3. Results

### 3.1. CB Progenitors and CB-Derived iPSCs Closely Resemble hESCs in DNA Repair Gene Expression Signature

Previous studies indicated that progenitor donor cells were more amenable than differentiated cells in reprogramming to a pluripotent state [32, 33]. We performed microarray-based analysis to determine the DDR gene expression profile of hiPSCs (Table S1) derived via different methods (Figure 1(a)(i)). We found that donor CD34^+^ CB progenitors cluster more closely with hESCs than adult fibroblasts (Ad.Fib) donors in baseline expression of DNA repair genes, including poly (ADP-ribose) polymerase 1-PARP1 (involved in single-strand break repair and DSB repair), XRCC5 (*a.k.a. *Ku80), and XRCC6 (*a.k.a.* Ku70) (involved in NHEJ DSB repair). Of note, expression of MYC and XRCC6 in CB progenitors was even higher than that for hESCs (Figure 1(a)(i)) [20]. Additionally, PARP1 and XRCC5 were expressed at higher baseline levels in sa-CB-iPSCs than in standard CB-iPSC lines (Figure 1(a)(ii)).

To determine whether the levels of expression of these repair gene transcripts translated into functional differences in protein levels, we performed immunoblot analyses on hiPSCs from these representative categories. Although steady state protein levels of ATM, Ku80, and PARP1 in sa-CB-iPSCs were similar to standard CB-iPSCs and hESCs, donor CD34^+^ CB progenitor baseline expression of these DNA repair proteins more closely resembled hESCs (^*∗*^ *p* < 0.05), compared to Ad.Fib (^*∗∗*^ *p* < 0.01 difference) (Figures 1(b)(i) and 1(b)(ii)). These results suggested that CD34^+^ CB progenitors may already possess hESC-like expression of DDR pathway components, even prior to initiation of reprogramming.

### 3.2. Sa-CB-iPSCs Resemble hESCs in Their DDR Response to Radiation

Irradiation (IR) elicits several posttranslational modifications of the components of DDR pathway. Irradiated hESCs and hiPSCs rapidly activate the ataxia telangiectasia and Rad3-related (ATR) and ataxia telangiectasia mutated (ATM) kinase-dependent DDR signaling [34], phosphorylating targets, such as p53 and H2AX [17, 35]. While ATR responds mainly to single strand breaks (SSBs) and stalled replication forks, ATM is activated in response to DSBs. Moreover, ATM deficiency confers hypersensitivity to IR [36].

**Figure 2:**
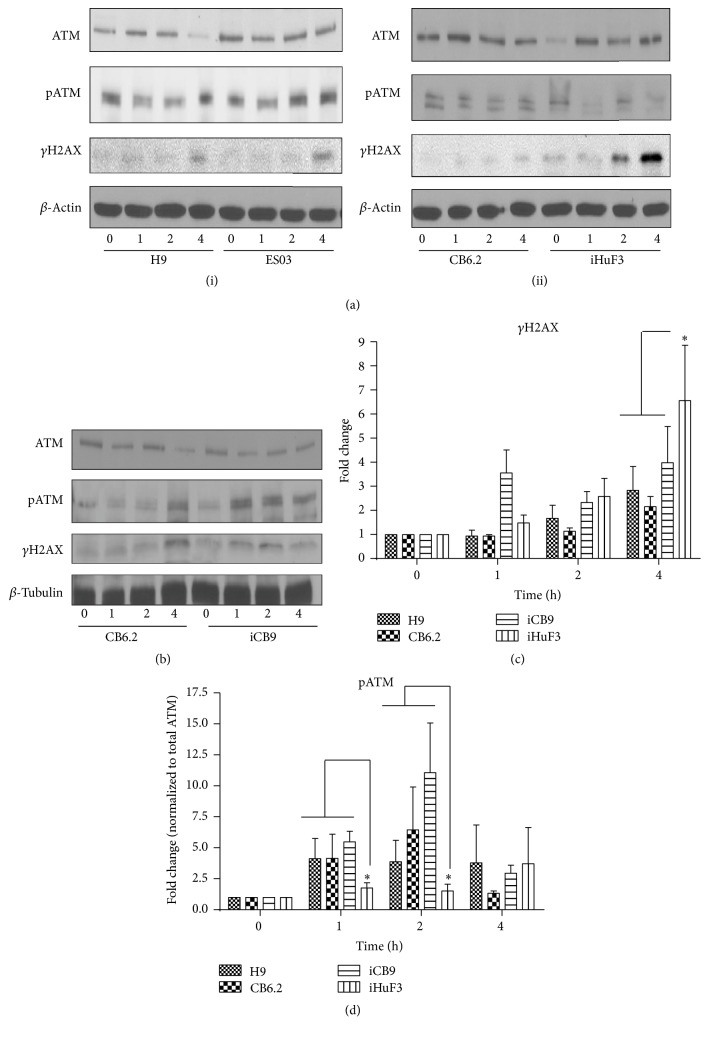
sa-CB-iPSCs closely resemble hESCs in DSB damage response to radiation. ((a)(i), (a)(ii), and (b)) Representative Western blot analysis depicting the expression of phosphorylated ATM (pATM) and H2AX (*γ*H2AX) in cell lysates from H9, ES03, CB6.2, iCB9, and iHuF3 at time 0 and at 1 h, 2 h, and 4 h after IR. *β*-Actin and *β*-tubulin were used as loading controls. Cells were exposed to IR (X-ray; 2 Gy) recovered at the indicated time points and immunoblotting was performed to analyze the kinetics of DDR protein expression. ((c) and (d)) Densitometry analysis of the Western blots for (c) *γ*H2AX and (d) pATM (normalized to total ATM), using ImageJ software. Statistical significance of the data was determined using 2-way ANOVA analysis with Bonferroni posttests to compare the replicates (three independent experiments). *γ*H2AX expression in iHuF3 is significantly different at 4 h compared to the following (versus H9 and CB6.2, ^*∗*^ *p* < 0.05). pATM expression in iHuF3 is significantly different at 1 h and 2 h, compared to all other cell lines (^*∗*^ *p* < 0.05).

To determine the efficacy of DDR, representative CB-derived hiPSCs (i.e., sa-CB-iPSC (CB6.2), standard CB-derived hiPSC (iCB9), and fibroblast -derived (iHUF3)) [34] were treated with IR (2 Gy) and compared with IR-treated hESCs (i.e., H9 and ESO3). To examine the DSB response in IR-treated hiPSCs, we performed immunoblotting for phosphorylation of H2AX on Ser139 (*γ*H2AX), which functions to assemble DSB repair factors [35]. In hESCs (H9 and ES03) and sa-CB-iPSC (CB6.2), *γ*H2AX expression was evident at 4 h after IR (Figures 2(a) and 2(c)), indicating activation of a DSB response. All tested hiPSCs exhibited kinetics of H2AX phosphorylation similar to hESCs (Figures 2(a)–2(c)). Interestingly, hESCs and hiPSCs did not differ significantly in the expression levels of total ATM protein (Figures 2(a) and 2(b)). Notably, hESCs and CB-derived hiPSCs, including sa-CB-iPSC (CB6.2) and standard CB-iPSC (iCB9), demonstrated activation of ATM via phosphorylation at Ser1981 up to 4 h following IR (Figures 2(a)(i), 2(a)(ii), 2(b), and 2(d)). Interestingly, in comparison to hESCs and CB-iPSCs, fibroblast-derived iHuF3 cells exhibited less robust phosphorylation of ATM following exposure to 2 Gy IR (^*∗*^ *p* < 0.05) (Figures 2(a), 2(b), and 2(d)).

**Figure 3:**
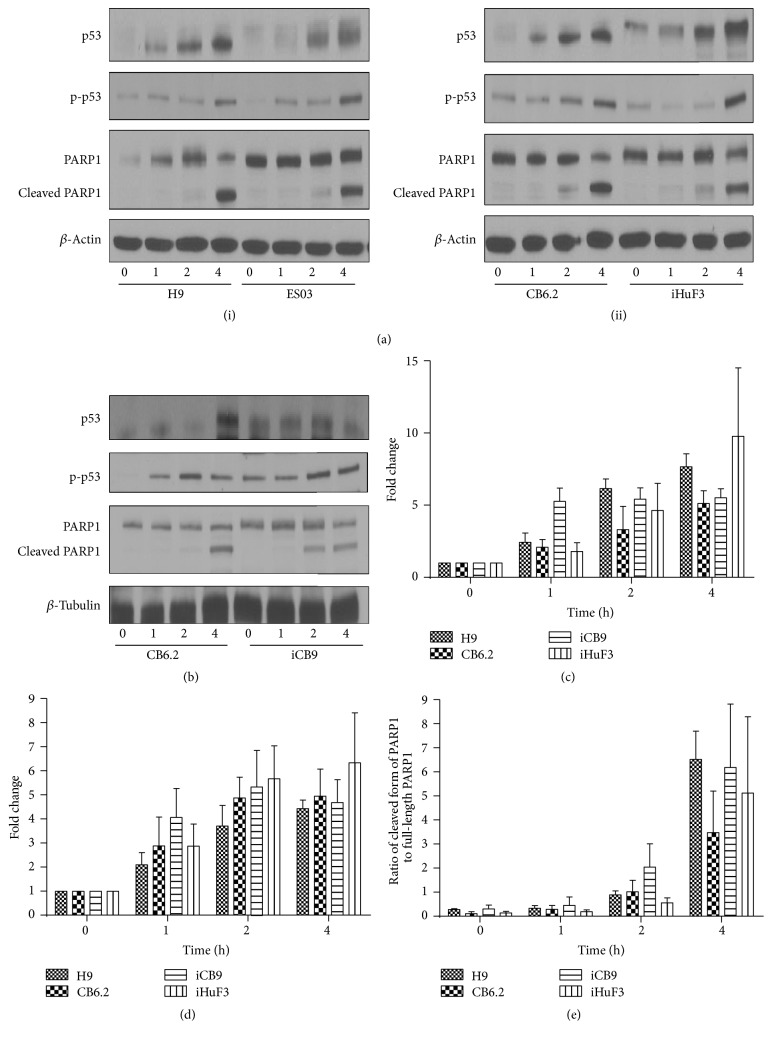
hESCs and hiPSCs have similar kinetics of apoptotic response to radiation exposure. ((a)(i), (a)(ii), and (b)) Representative Western blot analysis depicting the expression of p53 and PARP1 (full-length: 116 kDa; cleaved form: 89 kDa) in cell lysates from H9, ES03, CB6.2, iCB9, and iHuF3 at time 0 h and at 1 h, 2 h, and 4 h after IR. (c–e) Densitometry analysis of the western blots for measurement of (c) total p53, (d) phosphorylated p53^Ser15^ (p-p53), and (e) PARP1 cleavage, using ImageJ software. Statistical significance of the data was determined using 2-way ANOVA with Bonferroni posttests to compare the replicates (three independent experiments).

We next examined the activity of another ATM target, the tumor suppressor p53, whose expression is stabilized upon DNA damage, thus activating the DNA binding function of p53. Posttranslational modification of p53 via phosphorylation at Ser15 has been proposed to be an important mechanism by which p53 is stabilized and its functions are regulated [37]. However, phosphorylation is not an absolute necessity for DNA damage-induced stabilization of p53 [37]. Our results show that P53 activation, measured by monitoring total p53 protein and phosphorylation at Ser15, occurred with similar kinetics in all the hiPSCs and hESCs, with levels increasing between 0 and 4 h after IR (Figures 3(a)–3(c)). Moreover, standard hiPSC lines (e.g., iCB9 and iHuF3) consistently displayed higher baseline levels of total p53 protein in untreated controls, in comparison to hESC (H9), ESO3, and sa-CB-iPSC (CB6.2) (Figures 3(a)–3(d)). In our observation, activation of p53 in cells following IR is mostly contributed by the stabilization of total p53 protein, as the relative changes in levels of phosphorylated protein were insignificant when its expression is normalized to total p53 (except for CB6.2 (2 h), *p* < 0.05) (Figure S2).

We next investigated apoptotic responses of hiPSC cell lines to IR-induced damage. All pluripotent stem cells have been reported to exhibit hypersensitivity to radiation, with substantial cell death observed within 24 h after exposure to a low dose of ionizing radiation (1-2 Gy IR) [17, 20, 34]. We therefore reasoned that cells with higher levels of cytotoxic DSBs may induce apoptosis to avoid genotoxic stress. Using PARP1 cleavage as an apoptotic marker, IR-exposed cells were examined by immunoblotting. Notably, there were only subtle differences observed in the kinetics of PARP1 cleavage among hESCs and all hiPSCs. PARP1 was observed predominantly in the cleaved form 4 h after IR in all examined cell lines (Figures 3(a), 3(b), and 3(d)). These results indicated that despite the subtle differences in levels of DNA damage, reprogramming renders all hiPSCs equally hypersensitive to ionizing radiation-induced apoptosis.

### 3.3. Sa-CB-iPSCs More Closely Resemble hESCs in Nonhomologous End Joining (NHEJ) Repair

**Figure 4:**
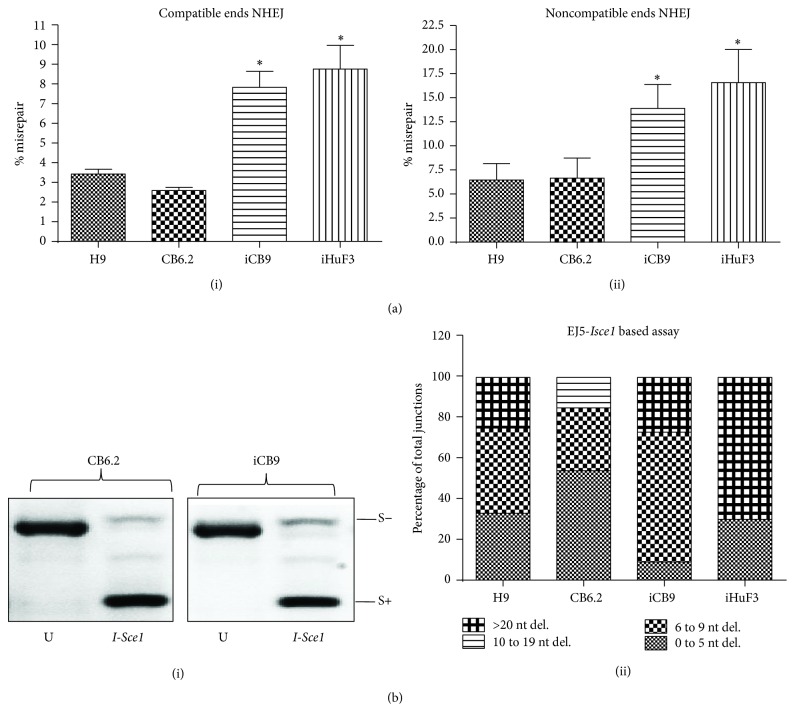
sa-CB-iPSC closely resembled hESC showing greater accuracy of nonhomologous end joining (NHEJ) repair. ((a)(i) and (a)(ii)) Analysis of repair products indicating percentage of misrepair in the* in vitro* PUC18-based end-joining assay. The misrepair % is calculated by dividing the total # of white colonies by total # of colonies, that is, blue + white, recovered from transformation of the repair products. (a)(i) demonstrates the % misrepair when the dialyzed nuclear lysates from respective cell lines are incubated with PUC18 linearized using* EcoR1*, giving compatible DNA ends; and (a)(ii) demonstrates the % misrepair when the dialyzed nuclear lysates from respective cell lines are incubated with PUC18 linearized using two restriction endonucleases (*Kpn1/SacI*), giving noncompatible DNA ends. Statistical significance of the data was determined using one-way ANOVA with Bonferroni posttests to compare all pairs of columns (cell lines). The data is significantly different for H9 or CB6.2 versus iCB9 or iHuF3 (^*∗*^ *p* < 0.05). (b)(i) Shown is a representative gel image of the PCR products from CB6.2 and iCB9 that are redigested with* I-Sce1 *or left uncut (U). All the S+ products on the gel represent correct repair that restores the* I-Sce1 *site in the plasmid. (S−) products represent the* I-Sce1 *resistant repair products, which were cloned into TOP10 competent cells. (b)(ii) The clones, each representing different repair products, were analyzed by sequencing across* I-Sce1* junction. Data represents ~10–15 clones analyzed in H9, CB6.2, iCB9, and iHuF3. The data is significantly different for iCB9 versus H9 and CB6.2 (0–5 nt/6–9 nt deletions) or iHuF3 versus H9, CB6.2, and iCB9 (>20 nt deletions) (^*∗*^ *p* < 0.05).

Differences in baseline levels of DNA damage markers between hiPSCs noted above may also be accounted for by differences in DSB repair [17]. For example, increased DSB formation could result from decreased efficiency of repair, which can lead to increased error-prone repair or misrepair. Thus, we next determined whether the CB.iPSCs derived with the same factors but using distinct episomal reprogramming methods demonstrated different DSB repair efficiencies. We employed an established end-joining plasmid-reactivation repair assay and observed that hESC H9 and sa-CB-iPSC CB6.2 displayed the lowest percentage of misrepair (approximately 3%). In contrast, standard hiPSCs iHuF3 and iCB9 possessed a significantly higher percentage of misrepair (approximately 8–12%; ^*∗*^ *p* < 0.05), when either compatible DSB ends or noncompatible DSB ends (which require additional processing steps in end joining) were used (Figures 4(a)(i) and 4(a)(ii)). To further confirm these results, we utilized an additional modified end-joining assay designed by Gunn and Stark [38] that measures DSB repair junctions representing repair of complementary or noncomplementary ends (Figure S1). We incubated* I-Sce1-*linearized pimEJ5GFP plasmid with nuclear extracts of pluripotent cell lines for measurement of* in vitro *plasmid reactivation (Figure 4(b)(i)), and the* I-Sce1* resistant fraction (“S−” products) was further analyzed for quantification and characterization of DNA deletions (Figure 4(b)(ii)). Sequencing of approximately 10–15 “S−” DNA clones recovered from end-joining experiments using H9 and CB6.2 extracts indicated that deletions in the DSB junctions were mainly in* I-Sce1* overhangs and were restricted to 1–5 nucleotides (nt) (33% and 54%, resp.). In contrast, only 1 out of 11 (9%) junctions that were recovered from iCB9 extracts showed 1–5 nt deletions (^*∗*^ *p* < 0.05, compared to H9, CB6.2), 64% of the junctions had 6–9 nt deletions, and ~27% of the junctions had >20 nt deletions. Strikingly, we observed that 70% of junctions (7 out of 10) recovered from iHuF3 had deletions >20 nt (^*∗*^ *p* < 0.05, compared to H9, CB6.2, and iCB9) (Figure 4(b)(ii)). This confirms that DNA end joining in sa-CB-iPSC CB6.2 more closely resembles that of hESCs and is less error-prone, compared to end-joining in the fibroblast-derived standard hiPSCs.

### 3.4. C-MYC Maintains the DDR and NHEJ in hESCs and Is Required for Less Error-Prone Repair in sa-CB-iPSCs

**Figure 5:**
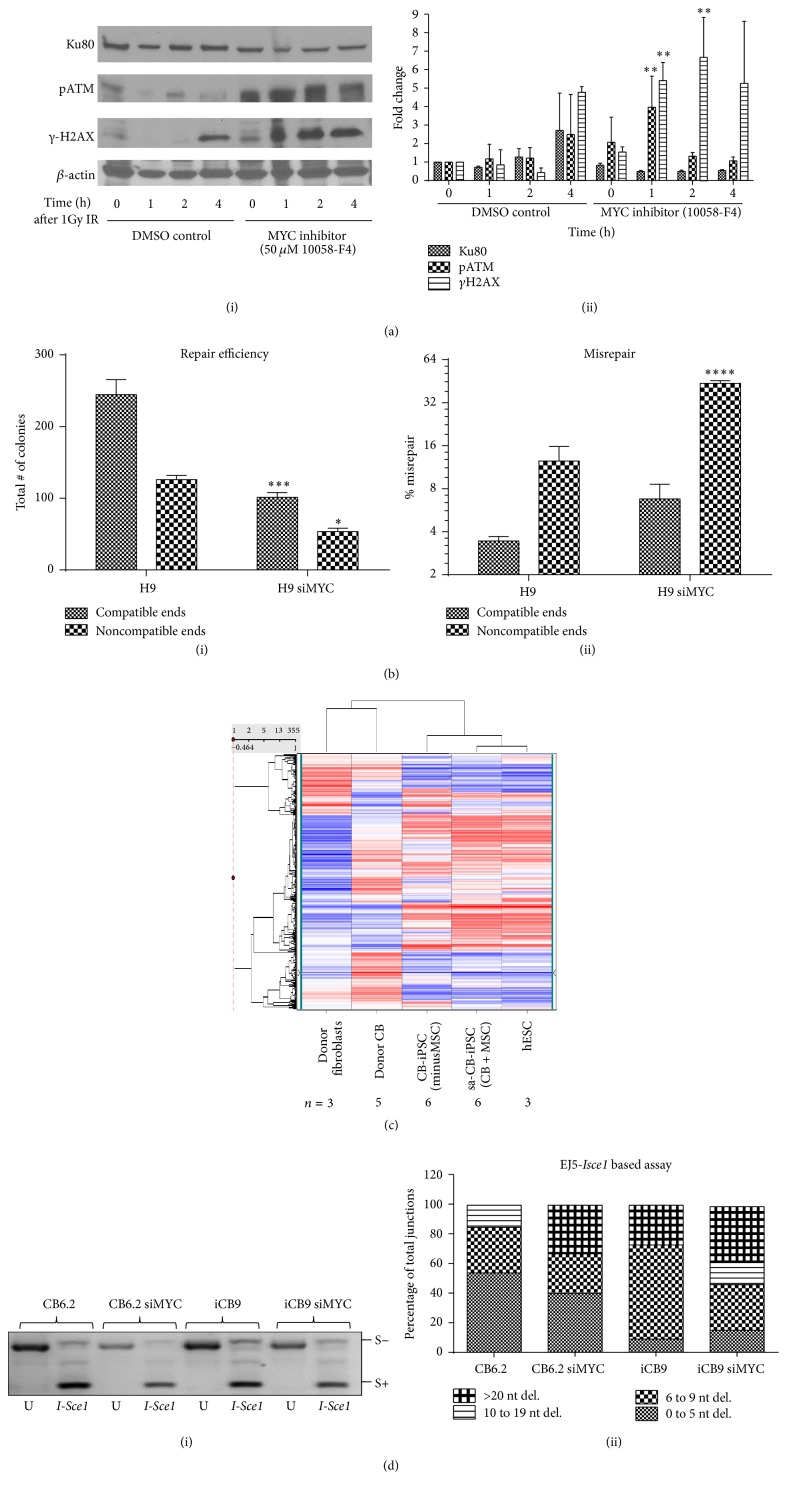
C-MYC maintained high-quality and high-efficiency NHEJ and is required for less error-prone DSB repair. (a)(i) Western blot analysis from whole cell extracts of H9 treated with either solvent control (DMSO) or MYC inhibitor (10058-F4) for 24 h at 50 *μ*M, exposed to IR (1 Gy), and collected at indicated time points. (a)(ii) Densitometry analysis comparing the means from three independent western blots as in ((a)(i)). Statistical significance of the data was determined using 2-way ANOVA with Bonferroni posttests (*γ*H2AX is significantly different between DMSO and MYC inhibition at 1 h, 2 h, and 4 h, *p* < 0.05; pATM is significantly different at 1 h, *p* < 0.05; Ku80 is significantly different at 0 h and 2 h, *p* < 0.05). ((b)(i) and (b)(ii)) The graph represents (i) efficiency of end-joining repair and (ii) percentage of misrepair in linearized PUC18 (with compatible ends) following incubation with extracts from H9 cells ± MYC siRNA. Repair efficiency is calculated by counting the total number of colonies (correctly repaired (blue) + incorrectly repaired (white)) from* in vitro *assays. Statistical significance was determined using paired *t*-test analysis (*p* < 0.01 between data sets H9 versus H9 siMYC). (c) Shown is the heatmap of log_2_ mean-subtracted normalized values of signal intensities from averaged independent biological replicate microarray samples (*n* = 3–6 per condition) representing the expression of genes in MYC module in mRNA from donor fibroblasts, donor CB (CD34^+^ population), and CB-iPSC lines (*i.e., *CB.iPS + MSC and CB.iPS (minusMSC)). ((d)(i) and (d)(ii)) (i) Shown is a representative gel image of the PCR products recovered from CB6.2 and iCB9 with or without treatments with siMYC. The PCR products are either redigested with* I-Sce1 *or left uncut (U). (S−) products represent the* I-Sce1 *resistant repair products. These (S−) fragments are cloned into TOP10 competent cells. (ii) The clones, each representing different repair products, were analyzed by sequencing near* I-Sce1* junction. Data represents ~10–15 clones analyzed in H9, CB6.2, iCB9, and iHuF3. The data is significantly different (^*∗*^ *p* < 0.05) for CB6.2 versus CB6.2 siMYC (>20 nt deletion) and iCB9 versus iCB9 siMYC (>20 nt deletion). Results are representative of the mean of two independent experiments of each set ± SEM; ^*∗*^ *p* < 0.05, ^*∗∗*^ *p* < 0.01, ^*∗∗∗*^ *p* < 0.001, and ^*∗∗∗∗*^ *p* < 0.0001, based on *t*-test analysis.

MYC modules, along with Core and Polycomb group genes, represent key gene circuits that contribute to the ES cell expression signature [39]. C-MYC depletion from the reprogramming cocktail significantly reduces the efficiency of reprogramming [40]. Interestingly, sa-CB-iPSCs were characterized by hESC-like MYC-regulated expression module and robustly expressed MYC complex genes [12]. In a different context, C-MYC has also been shown to regulate the transcription of several key DSB repair genes including Ku70 and BRCA1 in somatic cells [26]. Therefore, we questioned whether C-MYC contributes to enhanced efficacy and efficiency of repair in hESCs. As a proof of principle, C-MYC was depleted in hESC H9, using chemical inhibitor 10058-F4, which prevents MYC/MAX association and downstream signaling [41]. Following C-MYC inhibition (50 *μ*M, 24 h), the control and drug-treated cells were exposed to IR (1 Gy) and cells were examined at 0, 1, 2, and 4 hours after IR for expression of DDR proteins by immunoblotting. Notably, compared with untreated cells, C-MYC inhibition resulted in increased levels of *γ*H2AX 1 h after IR and persists until 4 h after IR (Figures 5(a)(i) and 5(a)(ii)). Whereas pATM expression changes after IR in untreated cells are more subtle, C-MYC inhibition results in persistence of pATM that decreases by 4 h. While Ku80 expression decreases with C-MYC inhibition, it is not significant compared with controls. These data suggest that C-MYC is involved in the radiation-induced DSB repair response in hESCs, facilitating repair.

We therefore next determined whether MYC inhibition reduced quality and efficiency of DNA end-joining in H9 cells. Remarkably, siRNA-mediated MYC knockdown (KD) in H9 resulted in a significant decrease in total NHEJ efficiency of these cells, as measured by counting total number of colonies (blue plus white) in an* in vitro* PUC18 assay (Figure 5(b)(i)). Moreover, we also observed a significant increase in the proportion of misrepaired colonies from hESC H9 cells treated with MYC siRNA (Figure 5(b)(ii)).

Since MSC activation of CB donors during reprogramming robustly activated MYC complex genes of pluripotency and facilitated high-capacity reprogramming of human MP differentiated from CD34^+^ cells [12], we sought to evaluate the MYC module expression networks in sa-CB-iPSC versus other hiPSC lines. Interestingly, microarray expression of MYC-regulated circuit genes in sa-CB-iPSC was more hESC-like relative to standard CB-iPSC (Figure 5(c)). We next determined whether inhibition of C-MYC affected the quality of end-joining in these categories of hiPSCs. For these experiments, we utilized* I-Sce1-*based assays (Figure S1) and measured DSB repair in these cells* in vitro *(see Section 2). As shown in Figure 5(d)(i), the majority of the GFP genes amplified from the PCR reaction were S+ (*I-Sce1 *sensitive), indicating that these extracts mostly produced distal-end joining products that are error-free. However, to determine the character of the errors from plasmid reactivation, PCR products resistant to* ISce1* restriction digestion (S−) were cloned into PCR2.1. Remarkably, similar to C-MYC depletion end-joining results in H9 (Figure 5(d)(ii)), analysis of DSB repair junctions indicated that the efficacy of DNA end-joining significantly deteriorated and became more error-prone when C-MYC was depleted in sa-CB-iPSC (CB6.2) (^*∗*^ *p* < 0.05) (Figure 5(d)(ii)). Specifically, while none of the 13 clones from WT CB6.2 had deletions of >20 nt, ~33% of clones (5 out of 15) showed deletions of >20 nt when C-MYC was depleted (^*∗*^ *p* < 0.05). Interestingly, in WT iCB9, 20% of clones had deletions of >20 nt nucleotides that further increased following C-MYC KD (38% versus 27% in WT) (Figure 5(d)(ii)). These results imply that C-MYC gene expression signature is linked to efficacious NHEJ DSB repair in pluripotent cells. Moreover, these data indicate that expression of C-MYC gene expression circuits in hiPSCs could be an important indicator of not only overall efficiency of reprogramming, but also overall DDR signaling and, in particular, repair of DSBs.

## 4. Discussion

Generating hiPSCs from adult cells represents one of the most exciting developments in regenerative medicine. However, potential clinical applications of hiPSCs are severely hampered by low efficiency of production and suboptimal genomic integrity. One study estimated that ~13% of hESC and hiPSC cultures demonstrated aberrant aneuploid karyotypes [42]. Comparative genomic analyses have revealed a high frequency of DNA copy-number variations (CNVs) in hiPSCs when compared to either hESCs or somatic cells of origin [9, 43]. DNA damage and inaccurate “follow-up” repair mechanisms likely present a significant source of genomic aberrations [44]. For example, reprogramming methods may introduce DNA lesions in the form of lethal DSBs [44]. DSB lesions are introduced by ectopic expression of reprogramming factors and appear to develop irrespective of the reprogramming methodology (i.e., integrative or nonintegrative) [44, 45]. DSB repair components also play an important role in controlling the efficiency of reprogramming [44, 46–50]. Cells that are impaired in HR genes, such as BRCA1/BRCA2 or NHEJ factor DNA ligase IV (LIG4), show significantly decreased capacity for reprogramming [44, 49]. However, it is not well understood whether the features that promote reprogramming further translate into hiPSCs with more robust and efficacious DSB repair properties.

Our study demonstrates that CB-iPSCs generated with high efficiency (sa-CB-iPSC) possess an hESC-like C-MYC transcript signature and have a DDR that more closely resembles hESCs, relative to hiPSCs derived via standard methods. Moreover, sa-CB-iPSCs also performed end-joining DSB repair with less errors, compared with standard CB.iPSCs. Notably, depletion of C-MYC led to increased end-joining errors, suggesting for the first time that MYC-regulated circuits may be required for maintaining genomic integrity in hiPSCs.

Cell differentiation leads to a decline in DNA repair capacity, which can further lead to accumulation of DNA damage and mutations [32, 33]. In contrast, stem progenitors possess greater overall capacity for efficient DNA repair. Stem-progenitor cells may also be more amenable to cellular reprogramming, compared with differentiated somatic cells [12, 51, 52]. However, sa-CB-iPSCs derived from human myeloid progenitors through MSC activation signals are generated even more efficiently (1–4%) and possess minimal interline variability when differentiated to vascular progenitors, compared with hiPSCs derived from CB mononuclear cells generated without MSC activation (0.2–0.3%) [52]. While no significant differences in baseline expression of mRNA transcripts and translated proteins for DDR genes were observed between CB-iPSCs derived via different methods, most significant differences emerged when these cells were analyzed for their DSB repair activities. sa-CB-iPSCs exhibited end-joining repair which was less error-prone and more closely resembles DSB repair properties in hESCs.

Repair of nonligatable ends by NHEJ requires an end-processing step for ligation and thus is prone to errors resulting in deletions of a few nucleotides at DSB repair junctions. IR damage induces NHEJ-mediated DNA misrepair events in late G2 cell cycle stage [53]. Interestingly, ATM suppresses genomic aberrations and incorrect end utilization during NHEJ, known as “distal-end joining,” formed as a consequence of multiple DSBs due to genotoxic stress [54, 55]. Although hESCs can uniquely employ high-fidelity NHEJ that can operate independently of ATM [56], hiPSCs perform error-prone DSB repair in particular when exposed to genotoxic stress [53]. Our studies indicate that despite similarities in levels of total ATM and ATM phosphorylation kinetics after IR, sa-CB-iPSCs and standard CB-iPSC have differences in NHEJ responses. In particular, standard fibroblast and CB-iPSCs demonstrated a higher percentage of large deletions (≥20 nt) in DSB junctions, compared to sa-CB-iPSCs and hESCs. Remarkably, “error-proneness” of NHEJ significantly escalates when pluripotent cells are subjected to IR stress under conditions of MYC inhibition.

MYC is an important regulator of transcription in hESCs and is one of the key factors employed in the generation of hiPSCs. Indeed, ectopic MYC is necessary for efficiently generating iPSCs [57, 58]. MYC interacts with the NuA4 complex, a regulator of ESC identity. and is the master regulator of a key ESC transcription program [14, 59, 60]. MYC also activates high telomerase activity during reprogramming via regulation of TERT [61]. Hematopoietic growth factor (GF) stimulation of myeloid progenitors differentiated from CD34^+^ CB cells activates C-MYC-regulated modules to hESC-like levels and facilitates their pluripotency induction [12]. These GF-activated progenitors robustly overexpress MYC complex genes, which have been found to be vital for pluripotency and facilitation of somatic reprogramming [12]. Interestingly, the C-MYC module signature in ESCs highly resembles the C-MYC module that is found in cancer cells [39]. Our data reveals that hESCs and sa-CB-iPSCs have a similar C-MYC module signature. Moreover, MYC inhibition results in more repair errors in hESC and hiPSCs. Thus, while NHEJ in somatic cells is considered error-prone [19], in normal pluripotent cells, C-MYC appears to be required for maintaining a more error-free NHEJ repair. Notably, putative C-MYC binding sites have been identified in the regulatory regions of several NHEJ genes, suggesting a potential mechanism through which C-MYC may maintain error-free NHEJ in hESCs and hiPSCs [26, 62].

In conclusion, our studies show that the various methods for generating hiPSCs may affect the pathways that regulate genomic integrity. Further characterization is required to determine how these pathways are interconnected and will enable improvement of the genomic integrity of hiPSCs. Knowing that C-MYC is also a master regulator of chromatin modifications [13, 60], its role in facilitating repair might be not only transcriptionally regulated but also epigenetically controlled. Thus, further elucidation of the role of C-MYC in maintenance of genomic integrity, regulating the balance between “good repair” and “bad repair” in pluripotent cells, is required.

## Supplementary Material

Supplementary Figure 1: Schematic of the I-Sce1 EJ5 plasmid reactivation assay. Analysis of the repair products from the *EJ5-I-Sce1*-based end-joining assay.pimEJ5GFP was linearized using *I-Sce1* and incubated with the dialyzed nuclear extracts from the respective cell lines. GFP genes were PCR amplified from the *in vitro *ligation reaction.
Supplementary Figure 2: Phosphorylated p53 normalization to total p53 protein. Densitometry analysis of the Western blots for measurement of phosphorylated p53^Ser15^ (p-p53) plotted relative to total p53 (alternate analysis of Fig 3C and Fig 3D), using ImageJ software. Statistical significance of the data was determined using 2-way ANOVA analysis with Bonferroni post-tests to compare the replicates (three-independent experiments).
