# Supplementary material for: High-Fidelity Reprogrammed Human IPSCs Have a High Efficacy of DNA Repair and Resemble hESCs in Their MYC Transcriptional Signature
Source: Stem Cells Int. 2016 Sep 1;2016:3826249. doi: 10.1155/2016/3826249 (PMC5023833; doi:10.1155/2016/3826249)
Supplement: Supplementary file 1 — Supplementary Figure 1: Schematic of the I-Sce1 EJ5 plasmid reactivation assay. Analysis of the repair products from the EJ5-I-Sce1-based end-joining assay.pimEJ5GFP was linearized using I-Sce1 and incubated with the dialyzed nuclear extracts from the respective cell lines. GFP genes were PCR amplified from the in vitro ligation reaction. Supplementary Figure 2: Phosphorylated p53 normalization to total p53 protein. Densitometry analysis of the Western blots for measurement of phosphorylated p53Ser15 (p-p53) plotted relative to total p53 (alternate analysis of Fig 3C and Fig 3D), using ImageJ software. Statistical significance of the data was determined using 2-way ANOVA analysis with Bonferroni post-tests to compare the replicates (three-independent experiments). [file 3826249.f1.pdf]

Supp. Fig. 1

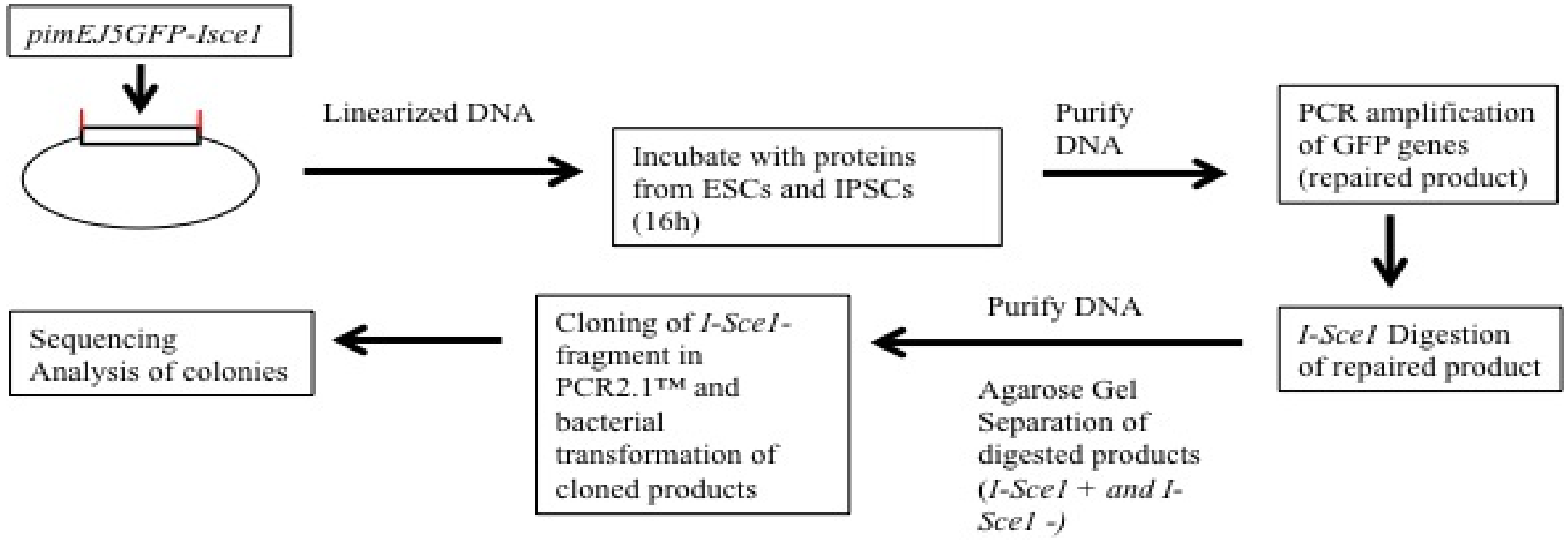

- *I-Sce1* + --------> *I-Sce1* sensitive => 'Good repair'
- *I-Sce1* - --------> *I-Sce1* resistant => 'Bad repair'

Supp. Fig. S2

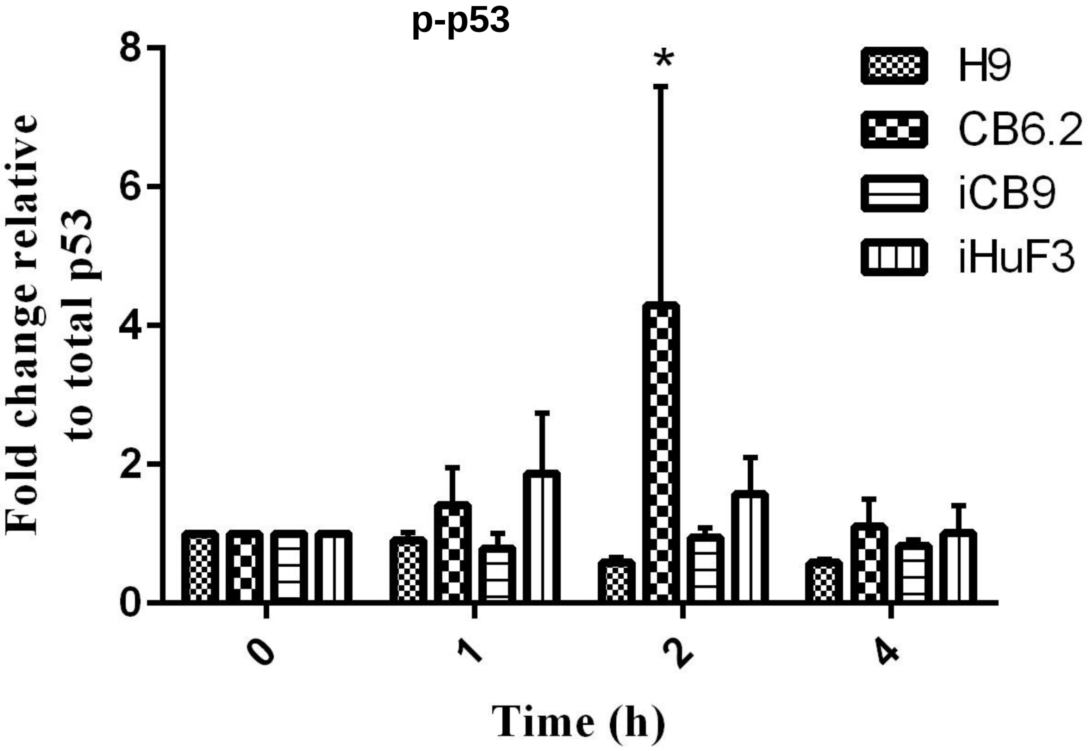
p-p53
Fold change relative to total p53
8
6
4
2
0
*
H9
CB6.2
iCB9
iHuF3
0
1
2
4
Time (h)

Table S1: Description of the cell lines used for the microarray analysis including the information about the passage numbers.

Class	Name	Passage	Long description
standard CB-iPSC	E17C1	p9	nonviral 4F non-stromal activated cord blood iPSC clone E17C1 p9
standard CB-iPSC	E20C2	p18	nonviral 4F non-stromal activated cord blood iPSC clone E20C2 p18
standard CB-iPSC	E24C1	p17	nonviral 4F non-stromal activated cord blood iPSC clone E24C1 p17
standard CB-iPSC	iCB2-5	p18	nonviral 7F non-stromal activated cord blood iPSC clone iCB 2-5 p18
standard CB-iPSC	iCB8	p19	nonviral 7F non-stromal activated cord blood iPSC clone iCB8 p19
standard CB-iPSC	iCB9	p18	nonviral 7F non-stromal activated cord blood iPSC clone iCB9 p18
sa-CB-iPSC	E5C3	p18	nonviral 4F stromal activated cord blood iPSC clone E5C3 p18
sa-CB-iPSC	E12C1	ND	nonviral 4F stromal activated cord blood iPSC clone E12C1
sa-CB-iPSC	E12C5	p19	nonviral 4F stromal activated cord blood iPSC clone E12C5 p19
sa-CB-iPSC	E17C6	p21	nonviral 4F stromal activated cord blood iPSC clone E17C6 p21
sa-CB-iPSC	6.2	p18	nonviral 7F stromal activated cord blood iPSC clone 6.2 p18
sa-CB-iPSC	6.13	p22	nonviral 7F stromal activated cord blood iPSC clone 6.13 p22
sa-CB-iPSC	19.11	p17	nonviral 7F stromal activated cord blood iPSC clone 19.11 p17
Fibroblast iPSC	iHUF3	p20	viral 4F Adult Fibroblast iPSC clone HUF3 p20
hESC	H7	p38	hESC (H7) p38
hESC	H9	p60	hESC (H9) p60
hESC	ESO3	p84	hESC (ESO3) p84
